# Epidemiology of lymphoid malignancies: last decade update

**DOI:** 10.1186/2193-1801-2-70

**Published:** 2013-02-26

**Authors:** Silvana Novelli, Javier Briones, Jorge Sierra

**Affiliations:** Hematology Service, Hospital de la Santa Creu i Sant Pau, Barcelona, Spain

## Abstract

**Background:**

The non-Hodgkin lymphomas are 12^th^ most prevalent cancers in Europe. No recent update in the epidemiology of these lymphomas has been performed in our country. We diagnosed 701 new lymphomas during the period beginning January 1, 2000 and ending December 31, 2009 in our center.

**Findings:**

The most frequent lymphoma was diffuse large B cell lymphoma, followed by follicular lymphoma and then classic Hodgkin’s disease. The male:female ratio is 1.2:1. Diagnosis by age showed that non-Hodgkin’s lymphoma is by far more frequent in the 61-80 years old patients. On the other hand, classic Hodgkin’s lymphoma is more frequent in the 20-40 years old population.

**Conclusion:**

Our results are very similar to those published by other centers in Europe and United States

## Introduction

Cancer is one of the leading causes of death worldwide and accounted for 7.6 million deaths (13% of all deaths) in 2008 according to the World Health Organization. The age-world-standardized incidence rate (per 100000) for non-Hodgkin lymphoma (NHL) and Hodgkin lymphoma (HL) are 6.7 and 2 respectively. The NHL is the 12^th^ most prevalent cancer in Europe (GLOBOCAN 2008).

Lymphoid malignancies compose a variety of different morphologic and clinical syndromes. Many publications affirm that incidence of lymphoid malignancies is increasing despite only a few reports have been performed using at least the 2001 WHO classification (Jaffe et al. 2001).

The later classification was the first to incorporate morphology, immunophenotype, cytogenetic and molecular features, clinical behavior, and some known aspects of etiology and pathogenesis into the definition of each disease subtype.

Even though, the current classification is the 2008 WHO classification where new entities are defined, and solutions for problematic categories are sought, these features do not interfere with a new epidemiological analysis.

The aim of this report is to update the frequencies of lymphoid malignancies in the past 10 years in our center in Barcelona, Spain.

## Material and method

### Materials

Our analysis considers the frequencies of all lymphoid neoplasms diagnosed during the 10-year study period beginning January 1, 2000 and ending December 31, 2009.

For each newly identified lymphoid neoplasm case, patient demographic data, including age and sex, and information on the tumor histologic type, primary site, and immunophenotype were collected.

Our Hospital is a university hospital, which has a referral population around 500000 patients. It is considered a highly qualified center in hematologic disorders.

Tissue samples were studied by histological examination of surgical biopsy from an accessible nodal site by the Pathology department. Diagnosis was established based on clinical information and cyto-morphological diagnostic criteria as recommended by the current World Health Organization Classification.

All patients signed consent forms before biopsies were taken and it included the use of their samples for investigation purposes.

### Statistical analysis

Data was analyzed using SPSS for MAC OS version 20.

The statistical methods applied include frequency counts and cross tabulations.

## Findings

We diagnosed 701 new lymphomas during the period beginning January 1, 2000 and ending December 31, 2009.

The frequency for each year is described in Table 1. We diagnosed a mean of 74 lymphomas per year. There was not any significant frequency variation between years.

**Table 1:** **Lymphoma frequencies by year of diagnosis**

Year of diagnosis	Frequency
2000	78 (11.1%)
2001	67 (9.6%)
2002	74 (10.6%)
2003	85 (12.1%)
2004	87 (12.4%)
2005	53 (7.6%)
2006	30 (4.3%)
2007	70 (10%)
2008	83 (11.8%)
2009	74 (10.6%)
Total	701

The most frequent lymphoma was diffuse large B cell lymphoma, followed by follicular lymphoma and then classic Hodgkin’s disease (Table 2). Inside the diffuse large B cell lymphoma group we included 5 post allogeneic hematopoietic stem cell transplantation lymphoma (EBV-related). None of these patients had previous history of lymphoma and these patients were the only with recent previous chemotherapy administration history. Excluding post transplant lymphoma, EBV positivity was present only in 2% of the cases. (1,2% were older than 60 years old)

**Table 2:** **Lymphoma’s frequencies**

Diagnosis	Frequency
Diffuse large B cell lymphoma	241 (34.4%)
Chronic Lymphocytic Leukemia/Small Lymphocytic Lymphoma	37 (5.3%)
Mantle Cell Lymphoma	29 (4.1%)
Follicular Lymphoma	127 (18.1%)
Splenic Marginal Zone Lymphoma	16 (2.3%)
Nodal Marginal Zone Lymphoma	6 (0,9%)
Extranodal Marginal Zone Lymphoma	51 (7.3%)
Burkitt's Lymphoma	21 (3%)
Plasmablastic Lymphoma	4 (0.6%)
Peripheral T cell lymphoma NOS	32 (4.6%)
Angioimmunoblastic T cell Lymphoma	2 (0.3%)
Hodgkin's Lymphoma - Classic	65 (9.3%)
non-Classic Hodgkin's lymphoma	6 (0.9%)
Mycosis Fungoides - Sesary Syndrome	32 (4.6%)
Cutaneous Anaplastic Large Cell Lymphoma	5 (0.7%)
Primary Mediastinal B Cell Lymphoma	3 (0.4%)
Anaplastic Large Cell Lymphoma, Alk-Negative	5 (0.7%)
Anaplastic Large Cell Lymphoma, Alk-Positive	5 (0.7%)
T/NK Lymphoma	2 (0.3%)
Adult T Cell Lymphoma	1 (0.1%)
MALT	3 (0.4%)
Waldenström macroglobulinemia	3 (0.4%)
Cutaneous B Cell Lymphoma	1 (0.1%)
Cutaneous T Cell Lymphoma	4 (0.6%)
Total	701

### Frequencies by sex

The number of lympholiferative disorders diagnosed in our center between January 2000 and December 2009 was 701, 382 males and 319 females. The male:female ratio is 1.2:1.

Only splenic and nodal marginal cell lymphomas were found to be more frequent in women. This difference was also present in follicular lymphoma but in a minor degree.

### Frequencies by age

**Figure 1:**
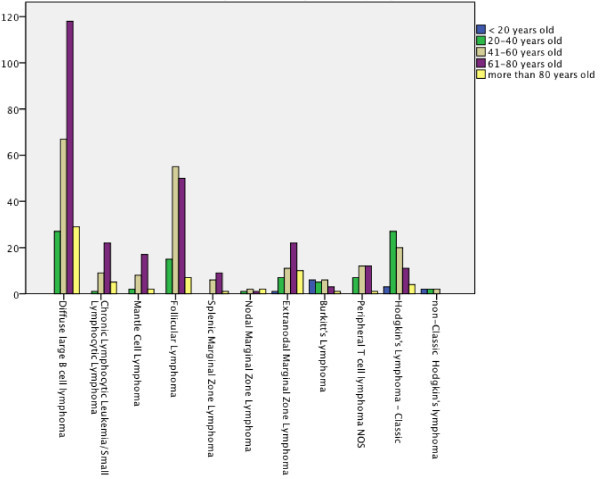
**Diagnosis by age.**

Diagnosis by age showed that non-Hodgkin’s lymphoma are by far more frequent in the 61-80 years old patients. On the other hand, Classic Hodgkin’s lymphoma is more frequent in the 20-40 years old population (Figure 1).

## Discussion

Despite recent advances in new therapeutic chemotherapy regimens for lymphoma, epidemiological studies worldwide are few and almost absent in Spain.

We considered very important to update the epidemiology of lymphomas in our center with the aim to focus our investigational efforts in the most prevalent type.

Our results are not so different from the other series published if we consider only the frequencies.

The European Cancer Registry-based project on hematologic malignancies (HAEMACARE) (Marcos-Gragera et al. 2011) includes only one Spanish region (Girone) with 1245 patients diagnosed in period of 8 years. If we extrapolate our data, which includes 701 new cases in a 10 year period for a referral population of 500000 habitants, we have a density of 140 new lymphoma cases per 1 million of referral population per year whereas in Girone they have an approximation of 178 new lymphoma cases per 1 million population per year. Comparing the frequencies of our data with the HAEMACARE data we have some differences within the B cell lymphomas. The most frequent B cell lymphoma in the HAEMACARE is CLL/SLL whereas for us is the DLBCL. The cause of this difference may be that in our data we only have included CLL/SLL cases without peripheral expression of the diseases (lymphocytosis). We also have excluded plasmacytoma and multiple myeloma.

There is a very similar work published by University of Benin Teaching Hospital (Nigeria) (Omoti et al. 2012) were they describe the frequencies in a five year interval. The differences between our results and theirs is the male:female ratio which is of 1.2:1 in our data and 1.6:1 in theirs. We also found some differences at the age of diagnosis, which was higher in our report (60-80 years old group). In Benin study the frequencies are higher at 46-59 years old group. Both discrepancies may be related to the “age” of general population and different sex distribution in both continents.

Regarding to the Asiatic reports (Yang et al. 2011) one of them shows higher frequencies in mature T and NK cell lymphomas (26% of total) than in our results in which they account less than 10% of lymphoid malignancies.

The US report (Morton et al. 2006) does not have differences with our frequencies. We have to say that is a remarkable study because of the quality of the data. They have collected data of 114548 lymphoma cases, included histologic subtypes, molecular data and wide demographic characteristics.

## Conclusion

We conclude that our results are very similar to those reported in other European countries and in the US but are some different from other continents. This suggests one more time that there is an etiologic heterogeneity among lymphomas and support the study of epidemiologic analysis by subtype.
